# Supplementary material for: Application of Synthetic Peptide CEP1 Increases Nutrient Uptake Rates Along Plant Roots
Source: Front Plant Sci. 2022 Jan 3;12:793145. doi: 10.3389/fpls.2021.793145 (PMC8763272; doi:10.3389/fpls.2021.793145)
Supplement: Supplementary Figure 1 — Dot plots showing nutrient uptake in 48 different plant roots treated with different peptides over 8 h as measured by the uptake platform. Nutrient depletion plots show raw data not normalized for root length. Uptake was measured for (A) nitrate, (B) phosphate, (C) sulfate, and (D) potassium. Each peptide and control had six replicates each. [file Image_1.pdf]

Supplementary figures

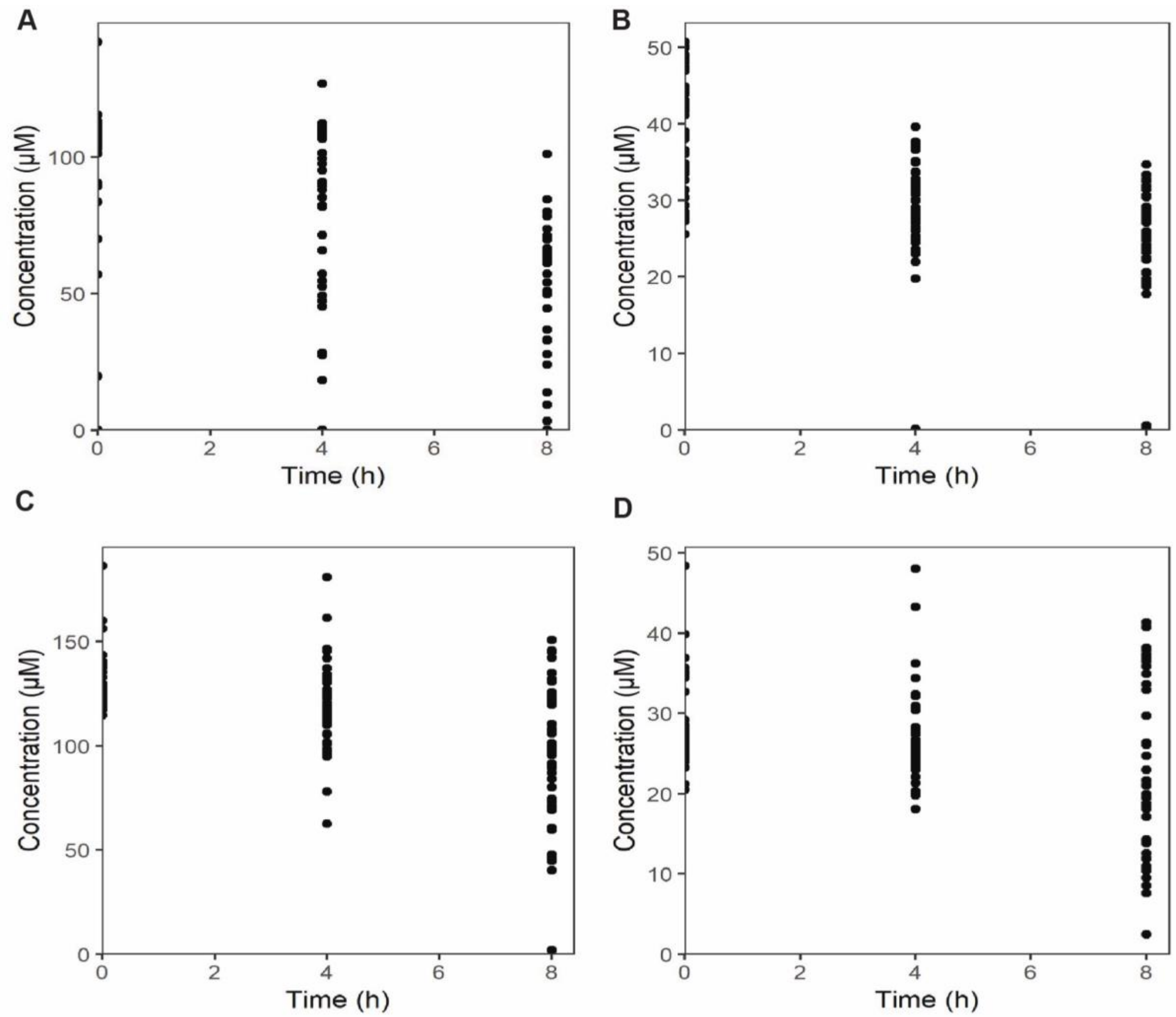

Figure S1. Dot plots showing nutrient uptake in 48 different plant roots treated with different peptides over 8 hours as measured by the uptake platform. Nutrient depletion plots show raw data not normalized for root length. Uptake was measured for A. Nitrate B. Phosphate C. Sulfate and D. Potassium. Each peptide and control had six replicates each.

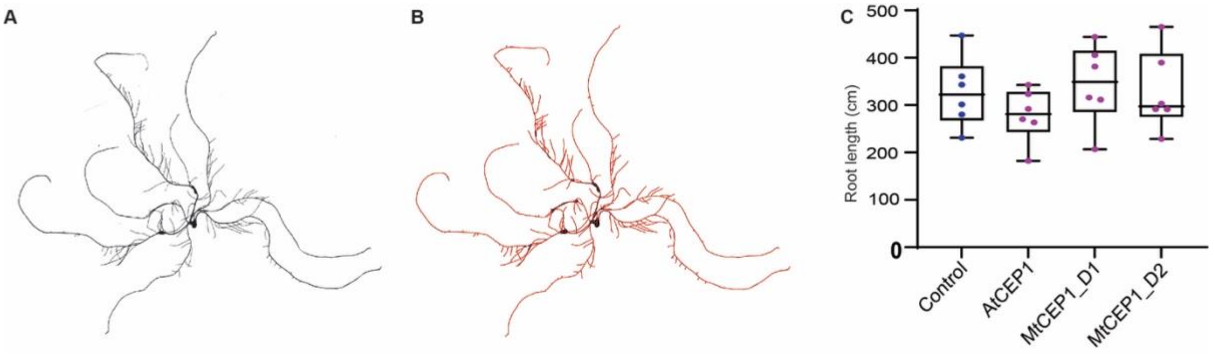

Figure S2. A. Rhizovision output showing representative root scan used for measuring total root length. B. RVExplorer output image to show an example segmentation of the root scan in A. C. Box plot showing difference in root length post treatment with peptides. No significant differences were found using a two-way ANOVA followed by a Dunnett's multiple comparison test.

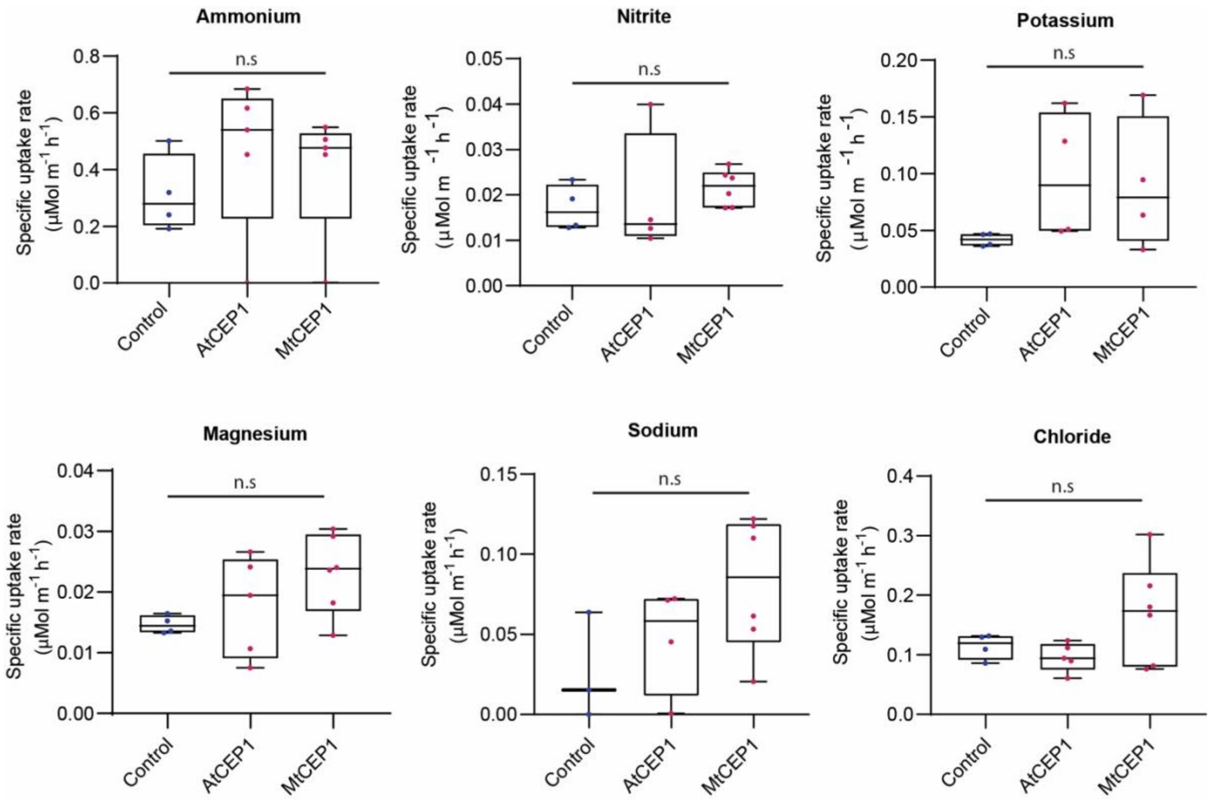

Figure S3. CEP1 has no significant effect on uptake of additional nutrients tested. Specific nutrient uptake rates of ammonium, nitrite, potassium, Magnesium, Sodium, Chloride as indicated in *M. truncatula* in the presence of the synthetic AtCEP1 peptide and MtCEP1D1 at a concentration of 1 µM. Student's t-test **p*<0.05, ***p*<0.01, ****p*<0.001. *n*=5-6 per treatment.

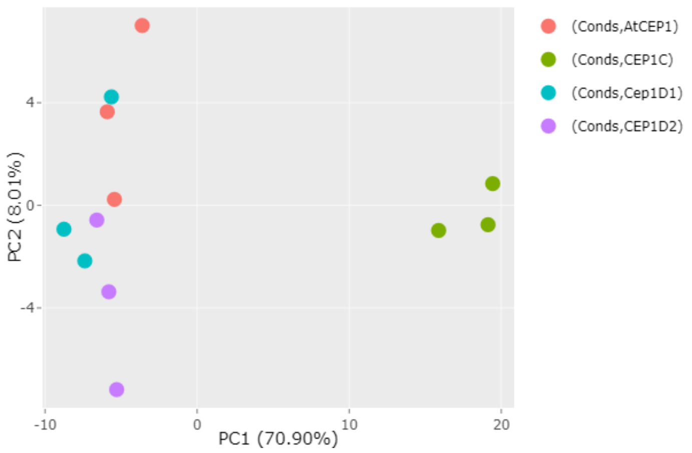

Figure S4: Principal component analysis graph showing distribution of biological replicates in peptide treated and control samples. Plot was generated using DESeq2 using a read count filter of 30 and a Median Ratio Normalization. Green dots represent the Control (CEP1C), Blue dots represent CEP1D1, Purple dots represent CEP1D2 and red dots represent AtCEP1 treatment.
